# Systematic review of guidance for the collection and use of patient-reported outcomes in real-world evidence generation to support regulation, reimbursement and health policy

**DOI:** 10.1186/s41687-022-00466-7

**Published:** 2022-06-02

**Authors:** Konrad Maruszczyk, Olalekan Lee Aiyegbusi, Barbara Torlinska, Philip Collis, Thomas Keeley, Melanie J. Calvert

**Affiliations:** 1 Centre for Patient Reported Outcome Research, Institute of Applied Health Research, University of Birmingham, Birmingham, UK; 2 Birmingham Health Partners Centre for Regulatory Science and Innovation, University of Birmingham, Birmingham, UK; 3 National Institute for Health Research (NIHR) Birmingham Biomedical Research Centre, University of Birmingham, Birmingham, UK; 4 NIHR Surgical Reconstruction and Microbiology Research Centre, University of Birmingham, Birmingham, UK; 5 NIHR Applied Research Collaborative West Midlands, University of Birmingham, Birmingham, UK; 6 GlaxoSmithKline (GSK), Patient Centered Outcome, Value Evidence and Outcomes, Brentford, UK

**Keywords:** PRO, RWE, Patient-reported outcomes, Real-world evidence, Guidelines, Recommendations

## Abstract

**Background:**

Real-world evidence (RWE) plays an increasingly important role within global regulatory and reimbursement processes. RWE generation can be enhanced by the collection and use of patient-reported outcomes (PROs), which can provide valuable information on the effectiveness, safety, and tolerability of health interventions from the patient perspective. This systematic review aims to examine and summarise the available PRO-specific recommendations and guidance for RWE generation.

**Methods and findings:**

Medical Literature Analysis and Retrieval System Online, Excerpta Medica Database, and websites of selected organisations were systematically searched to identify relevant publications. 1,249 articles were screened of which 7 papers met the eligibility criteria and were included in the review. The included publications provided PRO-specific recommendations to facilitate the use of PROs for RWE generation and these were extracted and grouped into eight major categories. These included: (1) instrument selection, (2) participation and engagement, (3) burden to health care professionals and patients, (4) stakeholder collaboration, (5) education and training, (6) PRO implementation process, (7) data collection and management, and (8) data analysis and presentation of results. The main limitation of the study was the potential exclusion of relevant publications, due to poor indexing of the databases and websites searched.

**Conclusions:**

PROs may provide valuable and crucial patient input in RWE generation. Whilst valuable insights can be gained from guidance for use of PROs in clinical care, there is a lack of international guidance specific to RWE generation in the context of use for regulatory decision-making, reimbursement, and health policy. Clear and appropriate evidence-based guidance is required to maximise the potential benefits of implementing PROs for RWE generation. Unique aspects between PRO guidance for clinical care and other purposes should be differentiated. The needs of various stakeholder groups (including patients, health care professionals, regulators, payers, and industry) should be considered when developing future guidelines.

**Supplementary Information:**

The online version contains supplementary material available at 10.1186/s41687-022-00466-7.

## Introduction

Real-World Evidence (RWE) is defined by the U.S. Food and Drug Administration (FDA) as clinical evidence assessing benefits and risks of a medical product derived from analysis of real-world data (RWD) [1]. RWE can be generated prospectively and retrospectively by different study designs [1]. RWD in turn is defined as “data relating to patient health status and/or the delivery of health care routinely collected from a variety of sources” [1]. The most common RWD sources are: electronic health records, claims databases, registries, and patient-generated data [1].

Currently, there is increasing recognition from global regulators, payers, and policy makers that patient-reported outcomes (PROs) — reports of health status directly provided by patients, without interpretation by a clinician or anyone else [2] — can provide valuable information on effectiveness, safety and tolerability from the patient perspective [3–6]. The U.S. FDA’s framework for Real-World Evidence Program acknowledged that PROs provide unique and valuable information which may complement the evidence obtained using traditional clinician-focused parameters [7]. The agency recently published its RWD draft guidelines on data sources, data standards, and regulatory considerations [8–11]. However, these guidelines make limited reference to PROs beyond referencing existing FDA 2009 guidance [12] and ensuring appropriate monitoring of the study, including where applicable, PROs.

It is also worth noting that PROs constitute a key part of U.S. Centers for Medicare & Medicaid Services Meaningful Measures Framework [13]. In the UK, the Medicines & Healthcare products Regulatory Agency (MHRA) recently issued two guideline documents focusing on the use of RWD to support regulatory decisions [14, 15]. The European Medicines Agency (EMA) currently uses RWE for safety monitoring and recently announced that the use of RWE will be established across its spectrum of regulatory use cases by 2025 [16].

Moreover, the recognition of the importance of PROs has led to a growing interest and increase in sponsorship by the pharmaceutical industry of real-world long-term safety studies which incorporate the longitudinal collection of PROs. Currently the PRO data for RWE generation are collected mainly in post-authorisation studies to support labelling claims, reimbursement and health policy making. For instance, the post-authorisation efficacy study for mepolizumab in the treatment of severe asthma [17] and post-authorisation efficacy and safety study for fingolimod in patients with relapsing–remitting multiple sclerosis [18] showed that the effectiveness of the drugs is consistent with clinical trial results under real-world settings.

In real-world contexts, prospective PRO collection has been limited and fragmented, with PROs collected in only 14% (8 out of 57) of recent post-authorization safety studies, consisting largely of one-off registries for post-marketing assessment sponsored by drug manufacturers in specific populations [19]. However, increasing collection of PROs in routine clinical care to support individual decision making and audit/benchmarking offers emerging opportunities to use the PRO data for multiple purposes including the assessment of real-world efficacy, safety, and tolerability of health interventions for regulatory, reimbursement and health policy purposes.

Several guidelines on the implementation of PROs exist but mainly focus on RCTs or clinical practice [5, 12, 20–25] and provide little or no recommendations for the use of PROs in the context of RWE generation, addressing the needs of regulators and policy makers. Therefore, the aim of this systematic review was to examine relevant literature and summarise PRO-specific recommendations for RWE generation to support regulation, reimbursement, and health policy, and highlight areas for future research.

## Methods

### Scope of the review

The review focused on PRO-specific recommendations for RWE generation. PROs were differentiated from other types of patient-reported or generated data, such as PREMs, unstructured patient-generated health data, patient-reported data about medication used, health care utilisation or events.

Studies were included if they provide recommendations for the use of PROs in RWE generation to support regulation, reimbursement, and health policy. No date limits or country restrictions were applied. In order to capture all available recommendations for PRO use in RWE generation, eligibility was not restricted to formally issued guidelines but also included any publications with recommendations or opinions on PROs in RWE generation including research, reports, discussion papers, books, commentary/opinion pieces and editorials.

Publications containing broad recommendations for PRO use only, e.g., general statements supporting PRO data collection in real-world setting or indicating the usefulness of PRO data, or highlighting the need for more patient-centric RWE research [8–11, 14–16, 26–29] were excluded. However, these were referenced in our discussions where appropriate.

Publications providing recommendations solely on the use of PROs in RCTs or to guide clinical care, and clinical RWE studies were excluded [23, 24, 30].

### Search strategy and publication selection

The systematic review was conducted according to a protocol registered in International Prospective Register of Systematic Reviews (PROSPERO), registration number: CRD42021235709. It was reported in compliance with the Preferred Reporting Items for Systematic Reviews and Meta-Analyses (PRISMA) guidelines [31] (see Additional file 1 for the completed PRISMA checklist). Medical Literature Analysis and Retrieval System Online (MEDLINE) and Excerpta Medica Database (EMBASE) were searched using broad search terms to identify relevant publications. The search was conducted using the controlled vocabulary and free text of the relevant databases. These included words related to “real-world evidence”, “patient-reported outcomes”, “guidelines” and “recommendations”. Moreover, the search terms used were adapted from published database search filters for “quality of life” [32] and “guidelines” [33]. No language or publication date restrictions were applied. For the full search strategy, see Additional file 2. Database searches were conducted on January 18, 2021. Two reviewers (KM, BT) independently screened the titles and abstracts according to the inclusion and exclusion criteria. Following this, the reviewers independently assessed the full texts of potentially relevant studies. At each stage, disagreements were resolved by discussion between the reviewers. If no consensus were reached, senior project members were consulted (MC, OLA). Records of screened entries, along with the reviewers’ reasons for inclusion and exclusion were held in EndNote X9 referencing software. When relevant conference abstracts were identified, we attempted to identify the full-text publication or conference output.

Other potentially relevant publications were identified from forward and backward citation searching of included studies. In addition, the grey literature was searched using a combination of the search terms from the original database search. Sources were:

- Google Scholar (100 first hits);
- HTA (Health Technology Assessment) agency websites: Canadian Agency for Drugs & Technologies in Health (CADTH), Haute Autorité de santé (HAS), The National Institute for Health and Care Excellence (NICE) and International HTA database, and NHS Evidence;
- Regulator websites: EMA and FDA;
- Professional organisations: Society for Health Economics and Outcomes Research (ISPOR), International Society for Quality of Life Research (ISOQOL), Standards in Analysing Patient-Reported Outcomes and Quality of Life Endpoints (SISAQoL) Consortium, Patient-Centered Outcomes Research Institute (PCORI), Agency for Healthcare Research and Quality (AHRQ), and International Society of Pharmacovigilance (ISOP).

### Data extraction

Relevant data were extracted into an Excel spreadsheet from the included publications by one reviewer (KM) and checked for accuracy (by BT). Data related to the following areas were extracted wherever possible: guidance issuing body, aim of the guidance, clinical area, patient population and recommended PRO instruments. Moreover, domains, described in the paper by Calvert et al. [6], were used as an initial framework for data extraction covering: objectives; patient population; instrument selection; frequency of administration; mode of administration; data collection method; data monitoring; presentation of results; ethics; data ownership and consent; audit; privacy; feedback to clinicians, patients, healthcare providers, drug manufacturers, regulatory authorities; and resources needed. Additional categories were added if identified information did not match any of the previously described domains. All extracted PRO-related recommendations were re-arranged into a smaller number of categories around similar issues addressed by the publications. Finally, these domains were grouped into major categories.

## Results

### Study selection

**Fig. 1:**
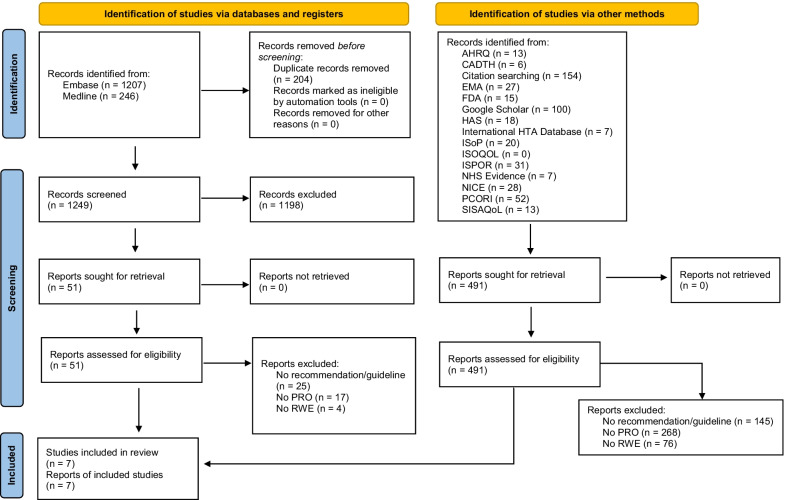
PRISMA flow diagram

The search strategy identified 1,453 potentially eligible entries, of which 1,249 remained after removing duplicates. After screening titles and abstracts, 1,198 entries were excluded, leaving 51 publications for full-text screening. Of these, five met the study inclusion criteria. An additional two entries were identified by reference and website searching, resulting in a total of seven publications included in the review. The PRISMA flow diagram (Fig. 1) provides an overview of the review process and study selection.

### Characteristics of included publications

The summary characteristics of all seven publications are presented in Table 1. Four were published in peer-reviewed journals [6, 34–36], two were conference posters [37, 38] and one was an online published report [39]. Four of the publications [6, 36, 37, 39] did not focus on a specific patient population or clinical area and provided recommendations applicable to the general patient cohorts. One of the publications focused on patients with dementia [34] and one gave recommendations focused on elderly patients [38]. One paper discussed PRO data collection among patients undergoing selected surgical interventions [35].

**Table 1:** Characteristics of individual studies

Author	Year	Issuer	Publication type	Patient population	Context for PRO usage	Aim of the publication	Scope of the publication
Hanson et al. [34]	2020	Division of Geriatric Medicine, University of North Carolina	Journal article	People with Dementia	Pragmatic Clinical Trials	To promote optimal use of outcomes relevant to people leaving with dementia and their caregivers in pragmatic trials	PCRO Core was proposed to promote optimal use of outcomes relevant to PLWD and their caregivers in pragmatic trials
Calvert et al. [6]	2019	CPROR, University of Birmingham	Comment	General	Drug development	To describe key challenges for use of PROs as part of RWE by payers and regulators	Overview of challenges in collecting, analysing, and integrating PRO data with other forms of RWE. Putting forward strategic priorities to help address these challenges
Rylands et al. [37]	2018	pH Associates (an OPEN Health company)	Conference poster	General	Research (various designs using RWD)	To summarise the key considerations for researchers collecting PRO data in RW studies	Summary of the key considerations for PRO data collection. Authors postulate the creation of a specific set of guidelines
Kyte et al. [35]	2016	CPROR, University of Birmingham	Journal article	Patients undergoing varicose vein, groin hernia and hip replacement surgery	Post-authorisation safety studies	To evaluate NHS PROMs programme for routine PRO data collection	Pointing areas for improvement in routinely collected PROs within NHS
Akiyama et al. [38]	2015	Bayer Yakuhin, Ltd	Conference poster	Elderly patients	Post-authorisation safety studies	To describe challenges and propose best practices for conducting post-marketing surveillance PRO surveys among elderly patients	A brief overview of challenges in collecting and using PRO data from elderly patients as part of post-marketing surveillance. Proposing best practices to help address these challenges
Banerjee et al. [36]	2013	PROSPER Consortium	Journal article	General	Safety reporting	To develop guidance on PRO-AE data, including the benefits of wider use and approaches for data capture and analysis. To support the wider use of PROs in safety reporting (pharmacovigilance)	Providing PRO-AEs taxonomy, suggesting a range of datasets that could be used for safety reporting, data collection mechanisms and analytical methodologies. Minimum core dataset for use by industry or regulators to structure PRO-AEs was proposed
ABPI [39]	2011	ABPI	Report	General	Research (various designs using RWD)	To provide further clarity around the definitions, use and practical issues which arise when undertaking RWD projects	Presentation of terminology related to RW studies. Methodological recommendations for RWD generation to be used for research, audit, and service evaluation purposes

*ABPI* The Association of the British Pharmaceutical Industry; *CPROR* Centre for Patient Reported Outcome Research; *NHS* National Health Service; *PCRO* patient- and caregiver-reported outcomes; *PLWD* people living with dementia; *PRO* patient-reported outcome; *PROM* patient-reported outcome measure; *RW* real-world; *RWD* real-world data; *RWE* real-world evidence

The included publications provided recommendations for PRO data collection and its use in different RWE settings. Two papers gave general recommendations relevant to real-world research [37, 39]. The remaining publications focused on: drug development [6], post-authorisation safety evaluation [35, 36, 38] and pragmatic clinical trials [34].

### Recommendations issued

The recommendations provided were grouped into eight major categories: (1) instrument selection, (2) participation and engagement, (3) burden to health care professionals (HCPs) and patients, (4) stakeholder collaboration, (5) education and training, (6) PRO implementation process, (7) data collection and management, and (8) data analysis and presentation of results.

An overview of the recommendation categories is presented in Table 2. Additionally, detailed data extracted from included studies for the major categories can be found in Additional file 3.

**Table 2:** Overview of recommendations categories

Recommendation categories	Hanson et al. [34]	Calvert et al. [6]	Rylands et al. [37]	Kyte et al. [35]	Akiyama et al. [38]	Banerjee et al. [36]	ABPI [39]
Measure selection	●	●	●	●	○	●	○
Participation and engagement	●	●	●	●	●	○	●
Burden to HCPs and patients	●	●	●	○	○	○	○
Stakeholder collaboration	○	●	○	○	○	○	○
Education and training	●	○	○	●	●	○	○
PRO implementation process	○	●	○	●	●	●	●
Data collection and management	●	●	●	●	●	●	●
Data analysis and presentation of results	○	●	○	●	○	●	●

● Includes, ○ Does not include.

#### Instrument selection

Five of seven included publications provided some level of advice about choosing appropriate PRO measure [6, 34–37]. PRO measure selection was discussed in the context of: instrument suitability for the target population, availability of relevant psychometric evidence supporting the use of PRO instrument in a given context and adaptation of existing instruments or development of new measures.

Calvert et al. [6] gave a broad recommendation stating that PROs measures used in the RWE setting need to be valid, consistent with the intended use and relevant to the identified needs of the target population. Banerjee et al. [36] proposed a core minimum dataset (including PROs) for non-regulated consumer websites listing information which should be collected from patients to allow for post-approval safety monitoring. Hanson et al. [34] highlighted the need for outcome measures to address patient or caregiver-centred outcome domains and to be acceptable to respondents.

The need for a definitive evidence base for PRO measures selected for use in a clinical setting was emphasized by Kyte et al. [35]. Hanson et al. [34] suggested that measure attributes such as psychometric properties (e.g. validity, reliability, sensitivity to change, floor/ceiling effect) should be considered when selecting PRO measures to identify instrument fit for purpose.

For situations where no appropriate measures are available, Hanson et al. [34] suggested the adaptation of existing measures or the development of de novo instruments. Particular attention was given to translation of existing questionnaires. Despite not recommending specific measures, authors often underlined the importance of using well translated PRO measures. Hanson et al. [34], Rylands et al. [37] and Calvert et al. [6] stressed the importance of adaptation and translation of PRO measures to ensure they match the literacy skills and are culturally relevant to diverse patient populations.

#### Participation and engagement

This category was split in two sub-domains. The first focuses on recommendations aiming to improve patient participation in a study and enhance quality of collected data. The second focuses on the involvement of different stakeholders in study design or conduct.

##### Study participation

Authors of four publications [6, 34, 37, 38] gave recommendations to strengthen patient participation in RWE studies. Calvert et al. [6] recommended to make questionnaires available in different languages to meet language requirements of diverse patient populations. Hanson et al. [34] stated that outcome measures used, should address patient or caregiver-centred outcome domain and be acceptable to respondents. Rylands et al. [37] noted that patient engagement and mode of recruitment strongly depend on the level of patient contact with healthcare services. Thus, it would be beneficial to consider the frequency of clinic visits required by patients when designing a study using RWD. Akiyama et al. [38] postulated that special attention is required at the participating sites for elderly patients. For example, large letters and simple wording may be helpful to be used for explanatory document and questionnaires dedicated for elderly patients. Also, posters and flyers may be used to promote the study.

##### Study development and conduct

Stakeholder involvement in designing RWE studies was recommended by five studies [6, 34, 35, 38, 39]. Greater HCP and health care providers involvement in planning study and data collection activities is beneficial. Akiyama et al. [38] noted the importance of involving clinicians with keen interest in PROs as it is key for successful data collection. Greater involvement of external stakeholders (payers, regulators, industry) in RWE studies can be obtained by demonstrating its benefits and importance to these organisations [34].

Hanson et al. [34] and Akiyama et al. [38] suggested to engage stakeholders early, particularly during PRO measure development process. Hanson et al. [34] focused mainly on collaboration with key stakeholders such as health system leadership. On the other hand, Akiyama et al. [38] and the ABPI [39] focused on both collaboration between internal (within industry or RWE study team) and external stakeholders (external experts, payers, regulators). Informing both internal and external stakeholders about justifications for PRO data collection for RWE and communicating to them the value of PRO assessments was also recommended [34, 38, 39].

Three publications stressed the importance of stakeholder involvement in PRO measure selection [6, 35, 38]. Focus groups and pilot tests were proposed as methods for enhancing stakeholder’s participation in measure selection or development.

#### Burden to HCPs and patients

The importance of not overburdening patients, clinicians and health care providers with frequent and lengthy data collections were described as key to the successful implementation of PRO measures for RWE generation. Hanson et al. [34] mentioned that paper questionnaires or patient interviews typically impose high respondent burden and are rarely tested in real-world clinical settings for wide-scale application to learn about patients’ experiences. Thus, computer adaptive testing, which may tailor PRO items to individual patient needs, may be considered to reduce patient burden [40, 41]. Two papers [6, 37] discussed the issue of patient burden and both postulated minimisation of patient, clinician, and health care provider burden by limiting frequency and complexity of data collection to a necessary minimum.

#### Stakeholder collaboration

Collaboration between relevant stakeholders was often mentioned as a key component for the successful use of PROs for RWE generation. According to Calvert et al. [6] international collaboration “…across multiple stakeholders including patients, caregivers, clinicians, regulators, ethicists, industry, payers and policy makers” is needed to establish a standardised approach to PRO assessment for RWE research. This multi-stakeholder collaboration is vital when collecting PRO data for multiple purposes to ensure that the data generated will meet their needs in the future.

#### Education and training

The importance of educating HCPs, patients, researchers, and other stakeholders on the potential benefits of PROs for RWE generation were mentioned by three publications [34, 35, 38]. Training focused on motivation maintenance and study procedures should be offered to HCPs involved [38]. Kyte et al. [35] recommended that efforts should be made to provide guidance to health care providers and patients on the interpretation and utilisation of benchmarks based on PROs captured in real world setting. Hanson et al. [34] created a searchable outcome measures library (including PRO measures) to educate other researchers interested in designing pragmatic trials in dementia.

#### PRO implementation process

Five publications [6, 35, 36, 38, 39] gave recommendations specific to the process of PRO implementation. Akiyama et al. [38] described the PRO inclusion process to collect data for post-marketing surveillance. They created a map that covers four stages: internal discussion, design and preparation, implementation, dissemination.

Calvert et al. [6] emphasised that special attention should be given to the resources needed to successfully implement PROs. Additional staff might be required to assist some of the patients with data collection. It is of paramount importance to secure up-front funding to cover costs associated with additional staff time needed, license fees for PRO measures, PRO training, data collection and devices costs. Kyte et al. [35] postulated that a shift to a “bottom-up” clinic-based PRO data collection approach that could be used for multiple purposes may be beneficial for patients and cost containment. Wider utilisation of data collected including post-marketing surveillance was postulated.

The implications of PRO data collection in real-world studies to address the legal requirement for obtaining Clinical Trial Authorisation and being compliant with the EU Clinical Trials Directive were mentioned by The Association of the British Pharmaceutical Industry (ABPI) guidance [39]. When PROs that are not in routine use are to be utilised to obtain data in RWE studies, legislation applicable to interventional clinical trials might need to be followed as PRO data collection can be seen as intervention administrated on the top of the regular care provision. Additionally, Banerjee et al. [36] advocated acceptance of non-medically confirmed adverse events reported by patients to account for more patient-centric approach in post-registration safety surveillance.

#### Data collection and management

Authors of all seven publications [6, 34–39] made recommendations for data collection and management. The following issues for RWE generation were specifically addressed: frequency of data collection, integration with other databases, data audit, data ownership, electronic data capture and impact of disease progression on data collection.

##### Frequency of data collection

As pointed out by Calvert et al. [6] frequency of data collection depends on stakeholder needs and the study population which should be considered early in study designing process. Additionally, patients with high symptom burden may require more frequent monitoring [6]. Two publications [6, 37] pointed out that the frequency of measurement is influenced by the schedule of patients’ visits and poses a challenge for data interpretation. Thus, appropriate methods of PRO measurement which facilitate data interpretation might be needed. Additionally, PRO data capture between scheduled visits could be considered. Calvert et al. [6] advocated the use of alert systems for PRO data collected between the visits, which would inform HCPs about issues requiring immediate attention. Additionally, reminders sent from electronic data capture systems may facilitate data collection and increase patient retention [38, 42].

##### Integration with other databases

Secondary data collection by integration of data capture with other databases, like electronic health records or registries, was suggested by two papers [6, 34]. Hanson et al. [34] pointed out that EHR systems might be used to facilitate PRO data collection if they had the capability to do so.

##### Data audit

The need for ongoing data quality audit was postulated by Calvert et al. [6]. Moreover, Rylands et al. [37] noted that potentially the amount of missing data, will be influenced by whether PRO data are routinely collected in clinical practice. Moreover, decisions about RWE study design (prospective or retrospective design) may be influenced by whether PROs are routinely collected or not.

##### Data ownership

Issues related to data ownership, storage and access were mentioned by four publications [6, 35, 36, 39]. It should be clearly stated who owns the rights to any data or potential intellectual property generated within the real-world study. Moreover, periods of data retention, entities responsible for their storage and applicable conditions need to be determined *a prior*i. Patients should be informed about the way their data will be used and they need to consent to that.

##### Electronic data capture

Five publications [6, 34–36, 38] provided recommendations specific to electronic data capture. All of them advocated the utilisation of electronic capture where appropriate. Akiyama et al. [38] maintained that electronic data collection is suitable for elderly patients and should be used wherever possible, as it streamlines data collection and improves quality of data collected. Electronic data capture can be conducted using the following devices: smartphone or website applications, automated interactive voice response telephone and wearable devices [34]. PRO-enabled website-based platforms were pointed as a preferable data source for collecting information from patients about treatments’ safety due to the higher quality of data captured [36]. However, the target population’s level of IT literacy should be considered when deciding on the mode of questionnaire administration as this can have serious implications for the representativeness of collected data [6]. Remote delivery of electronic PROs may lead to inequitable access if a substantial proportion of the target population have limited or no access to the internet. These issues could potentially decrease the value of PRO data collected as part of the RWE generation for regulatory purposes and may not be representative. Patients should be provided alternative modes of data collection (e.g., paper questionnaires, automated telephone services).

##### Impact of disease progression on data collection

Three publications [34, 37, 38] commented on changing patient health status over time, and its impact on data collection activities. Hanson et al. [34] highlighted that people living with dementia early in the disease trajectory can self-report. Nevertheless, once the disease progresses there may be a need for transition to proxy reporting, yet no best practices exist for interpretation of data reported by proxy. Similar concerns in the context of elderly patients were expressed by Akiyama et al. [38] Rylands et al. [37] acknowledged that patients’ ability to self-report need to be assessed early at the stage of study design.

#### Data analysis and presentation of results

Four publications [6, 35, 36, 39] provided guidance about results presentation and interpretation. Calvert et al. [6] and Kyte al. [35] provided general recommendations, claiming that data should be analysed and reported appropriately, according to the study objectives and the measure recommendations, following a methodologically robust process. Potential sources of bias and confounding need to be investigated and researchers could offer guidance on how to interpret and utilise findings. Guidance by ABPI [39] stressed how important it is to use sound methods for data generation, cleaning and analysis. Banerjee et al. [36] proposed suitable statistical methods for the analysis of datasets containing information about adverse events, such as appropriate descriptive statistics, methods to address disproportionality of results and multivariate analysis.

## Discussion

This review provides the first summary of available guidance for the use of PROs in RWE generation to support regulation, reimbursement, and health policy. Available guidance is fragmented, and it is evident that a better understanding of how to optimally collect and utilise PROs for RWE generation is needed. The main themes generated from the analysis of the included publications addressed issues relating to PRO data collection, analysis, and stakeholder collaboration.

It was recommended that steps should be taken to minimise the burden of PRO data collection on HCPs and patients, [6, 37] reduce data collection errors, allow automatic score calculations, improve data security, and speed up data collection process through the electronic data capture. These would enhance the quality of PROs obtained as part of RWD [23].

Statistical methods for the analysis of collected PRO data were also recommended [36]. Nevertheless, recommendations related to data analysis strategies to manage bias and confounding were not identified as part of this review. The need to develop such guidance seems evident. While existing PRO datasets collected in a real-world setting can be used to inform regulatory or reimbursement processes, a tailored approach to PRO data analysis is key to eliminating biases and confounding. Data captured in the real-world setting might require some additional statistical manipulation to account for inequitable access to PROs (e.g. due to low IT literacy among some groups of patients).

Our review highlighted the need for stakeholders’ engagement for successful PRO implementation. To improve efficiency of data collection activities for RWE, collaboration between different stakeholders need to be developed. Each stakeholder might have different expectations from the data collected as they can be used for various purposes. Thus, involvement of various stakeholders early at the stage of research planning is vital. To fully harness the potential benefits of collecting PRO data as part of real-world studies, it was recommended that various issues around stakeholder involvement, instrument selection and implementation need to be resolved [6, 34–38].

The potential benefits of collecting PROs may be maximised by using the data for multiple purposes including trials, routine care, audits, benchmarking and RWE generation [43]. For instance, in routine clinical practice, changes in an individual patient’s health status as indicated by their PRO data could facilitate the tailoring of their clinical management, which may, in turn, improve treatment outcomes. The utilisation of PRO alerts informing clinicians about changes in patients’ health status may lead to improvement in patient care by providing the opportunity for timely interventions (e.g. earlier clinic appointments or immediate hospitalisation) [43]. The same data can be aggregated for patients within healthcare systems to provide RWE of the safety and tolerability of health interventions. The use of PROs for multiple purposes would require agreement on the measures to be used to meet both regulatory and clinical needs. Feasibility of using the same PRO measures for multiple purposes could be explored further in the future research but it seems to be possible when focusing on aspects such as proximal symptoms and treatment tolerability.

The collection of PRO data in RWE research can bring numerous benefits by providing evidence of long-term safety, tolerability, and effectiveness from the patient perspective. The usefulness of longer-term additional data collection for the purpose of pharmaceutical licensing was previously described in article series by London School of Economics, which considered the use of RWE in Europe [44]. Additionally, the value of data reported directly by patients was evidenced by a comparison of chemotherapy-related adverse events reported by patients and clinicians, where patients tended to self-report more frequent and higher levels of symptoms than clinicians [45].

Although every effort was made to find potentially relevant publications (forwards/backwards citation searches, hand reference list searches, grey literature searches, and website searches were conducted) there is a possibility that some relevant publications were not identified due to poor indexing of the databases and websites searched. A limitation of this work was the dearth of guidance for the use of PROs in RWE to support regulation, reimbursement, and health policy. Even when recommendations were made, in some instances there were limited details on the rationale behind them.

The development of further guidance specific to PROs in RWE generation to support regulation, reimbursement and health policy will be an important next step. In doing so, it is of crucial importance to learn more about the various stakeholders’ needs and the current use of PROs in RWE generation to inform the guideline development. Patients, HCPs, regulators, payers, health care providers and industry will bring important perspectives about the specific needs of all those groups. The ISPOR Special Interest Group for Clinical Outcome Assessment is currently working on the standardisation of outcomes for real-world studies. Nevertheless, further research is needed to better inform the development of methodological recommendations for PRO-specific data generation as part of RWE for regulatory, reimbursement, and health policy.

## Conclusion

PROs may provide a valuable source of information in RWE generation from the patient perspective. Whilst valuable insights can be gained from guidance for use of PROs in clinical care, there is a lack of international guidance specific to RWE generation in the context of use for regulatory decision-making, reimbursement, and health policy. Clear and appropriate guidance, developed based on evidence, is required to maximise the potential benefits of implementing PROs for RWE generation. Unique aspects between PRO guidance for clinical care and other purposes should be differentiated. This review summarises some recommendations to optimise the use of PROs for RWE generation and highlights the need for further PRO-specific international guidelines to facilitate RWE generation for regulatory, reimbursement, and health policy. The needs of various stakeholder groups (including patients, health care professionals, regulators, payers, and industry) should be considered when developing future guidelines.

## Supplementary Information

**Additional file 1: **PRISMA 2020 checklist

**Additional file 2: **Search strategy

**Additional file 3: **Data extraction

## Data Availability

All data generated during this study are included in this published article and its supplementary information files. The record of inclusion/exclusion choices is available upon request.

## Abbreviations

ABPI: Association of the British Pharmaceutical Industry
AHRQ: Agency for Healthcare Research and Quality
CADTH: Canadian Agency for Drugs & Technologies in Health
EMA: European Medicines Agency
EMBASE: Excerpta Medica Database
FDA: U.S. Food and Drug Administration
HAS: Haute Autorité de Santé
HCP: Health care professionals
HTA: Health technology assessment
ISOP: International Society of Pharmacovigilance
ISOQOL: International Society for Quality of Life Research
ISPOR: Society for Health Economics and Outcomes Research
MEDLINE: Medical Literature Analysis and Retrieval System Online
MHRA: Medicines & Healthcare Products Regulatory Agency
NICE: National Institute for Health and Care Excellence
PCORI: Patient-Centered Outcomes Research Institute
PREMs: Patient-reported experience measures
PRISMA: Preferred Reporting items for systematic reviews and meta-analyses
PROs: Patient-reported outcomes
PROSPERO: International prospective register of systematic reviews
RCT: Randomised control trial
RWE: Real-world evidence
SISAQoL: Standards in Analysing Patient-Reported Outcomes and Quality of Life Endpoints
