# Supplementary material for: Systematic review of guidance for the collection and use of patient-reported outcomes in real-world evidence generation to support regulation, reimbursement and health policy
Source: J Patient Rep Outcomes. 2022 Jun 2;6:57. doi: 10.1186/s41687-022-00466-7 (PMC9163278; doi:10.1186/s41687-022-00466-7)
Supplement: Supplementary file 2 — Additional file 2: Search strategy [file 41687_2022_466_MOESM2_ESM.docx]

**Additional file 2. Search strategy**

## Inclusion/exclusion criteria

| Criteria | Inclusion | Exclusion |
| --- | --- | --- |
| Research area | Real-world data/evidence/research | Clinical setting |
| Outcome | - PRO | Other types of outcomes |
| Study type | - Guidelines - Recommendations | Other types of studies |
| Date | No limit | None |
| Countries | All | None |
| Publication type | Full research reports in journals, reports, discussion papers and books, commentaries, editorials | Letters, notes, news (publication type) |
| Language | English language studies^a^ | Non-English language studies |

^a^ English abstracts of non-English language studies will be considered for inclusion

## Search strategy

## Medline search terms (Searched on 18/01/2021)

| # | Criteria | Search term | Hits |
| --- | --- | --- | --- |
| 1 | Research area | real-world.ab,kf,kw,ti. | 30509 |
| 2 |  | RWE.ab,kf,kw,ti. | 172 |
| 3 |  | exp Pragmatic Clinical Trials as Topic/ | 560 |
| 4 |  | OR 1-3 | 31012 |
| 5 | Outcome | exp Health Status Indicators/ | 313292 |
| 6 |  | exp Health Status/ | 350958 |
| 7 |  | exp "Quality of Life"/ | 202456 |
| 8 |  | exp "Severity of Illness Index"/ | 258115 |
| 9 |  | exp Self-Assessment/ | 12664 |
| 10 |  | (self-report$ or self report$).ab,kf,kw,ti. | 136939 |
| 11 |  | functional.ab,kf,kw,ti. | 1068421 |
| 12 |  | patient reported.ab,kf,kw,ti. | 27488 |
| 13 |  | OR 5-12 | 1771468 |
| 14 |  | outcome$.ab,kf,kw,ti. | 1512012 |
| 15 |  | experience$.ab,kf,kw,ti. | 947903 |
| 16 |  | measure$.ab,kf,kw,ti. | 2852540 |
| 17 |  | assess$.ab,kf,kw,ti. | 2621781 |
| 18 |  | (score$ or scoring).ab,kf,kw,ti. | 841557 |
| 19 |  | index.ab,kf,kw,ti. | 674277 |
| 20 |  | indices.ab,kf,kw,ti. | 136671 |
| 21 |  | scale$.ab,kf,kw,ti. | 668812 |
| 22 |  | monitor$.ab,kf,kw,ti. | 703154 |
| 23 |  | OR 14-22 | 7243628 |
| 24 |  | 13 AND 23 | 886956 |
| 25 |  | exp Patient Reported Outcome Measures/ | 7234 |
| 26 |  | (qol or 'health-related quality of life' or 'hrqol' or 'quality of life' or 'nasal symptoms' or rhinitis or wpai or 'work loss' or 'opportunity loss' or productivity or depression or anxiety or 'global impression' or sleep or insomnia or 'burden of illness' or 'impact of disease' or 'patient based outcome' or 'patient experience' or 'patient perception' or 'patient relevant outcome' or 'patient-reported outcome' or 'patient reported outcome*' or 'pro' or 'attitude' or 'patient satisfaction' or 'preference' or 'satisfaction' or 'treatment attitude' or 'treatment importance' or 'treatment priorit*' or 'treatment perception').ab,kf,kw,ti. | 1205425 |
| 27 |  | OR 24-26 | 1854966 |
| 28 | Study type | exp Consensus/ | 14149 |
| 29 |  | exp Consensus Development Conference/ | 12171 |
| 30 |  | exp Guideline/ | 34166 |
| 31 |  | exp Practice Guideline/ | 27159 |
| 32 |  | exp Health Planning Guidelines/ | 4120 |
| 33 |  | exp Practice Guideline as Topic/ | 121541 |
| 34 |  | (guideline or practice guideline or consensus development conference or consensus development conference, NIH).pt. | 43688 |
| 35 |  | (position statement* or policy statement* or practice parameter* or best practice*).ti,ab,kf,kw. | 28140 |
| 36 |  | (standards or guideline or guidelines).ti,kf,kw. | 97563 |
| 37 |  | ((practice or treatment* or clinical) adj guideline*).ab. | 34714 |
| 38 |  | (CPG or CPGs).ti. | 5337 |
| 39 |  | consensus*.ti,kf,kw. | 22750 |
| 40 |  | recommendat*.ti,kf,kw. | 37235 |
| 41 |  | OR 28-40 | 299125 |
| 42 | N/A | 4 AND 27 AND 41 | **246** |

## Embase search terms (Searched on 18/01/2021)

| # | Criteria | Search term | Hits |
| --- | --- | --- | --- |
| 1 | Research area | real-world.ab,kw,ti. | 79881 |
| 2 |  | RWE.ab,kw,ti. | 830 |
| 3 |  | exp pragmatic trial/ | 1034 |
| 4 |  | OR 1-3 | 80996 |
| 5 | Outcome | exp Health Status Indicator/ | 32453 |
| 6 |  | exp Health Status/ | 249609 |
| 7 |  | exp "Quality of Life"/ | 516418 |
| 8 |  | exp "Severity of Illness Index"/ | 18180 |
| 9 |  | exp self evaluation/ | 32895 |
| 10 |  | (self-report$ or self report$).ab,kw,ti. | 216858 |
| 11 |  | functional.ab,kw,ti. | 1604037 |
| 12 |  | patient reported.ab,kw,ti. | 67216 |
| 13 |  | OR 5-12 | 2498755 |
| 14 |  | outcome$.ab,kw,ti. | 2731006 |
| 15 |  | experience$.ab,kw,ti. | 1560392 |
| 16 |  | measure$.ab,kw,ti. | 4409929 |
| 17 |  | assess$.ab,kw,ti. | 4441189 |
| 18 |  | (score$ or scoring).ab,kw,ti. | 1602984 |
| 19 |  | index.ab,kw,ti. | 1183191 |
| 20 |  | indices.ab,kw,ti. | 204115 |
| 21 |  | scale$.ab,kw,ti. | 1158398 |
| 22 |  | monitor$.ab,kw,ti. | 1161844 |
| 23 |  | OR 14-22 | 11704957 |
| 24 |  | 13 AND 23 | 1319671 |
| 25 |  | exp patient-reported outcome/ | 27724 |
| 26 |  | (qol or 'health-related quality of life' or 'hrqol' or 'quality of life' or 'nasal symptoms' or rhinitis or wpai or 'work loss' or 'opportunity loss' or productivity or depression or anxiety or 'global impression' or sleep or insomnia or 'burden of illness' or 'impact of disease' or 'patient based outcome' or 'patient experience' or 'patient perception' or 'patient relevant outcome' or 'patient-reported outcome' or 'patient reported outcome*' or 'pro' or 'attitude' or 'patient satisfaction' or 'preference' or 'satisfaction' or 'treatment attitude' or 'treatment importance' or 'treatment priorit*' or 'treatment perception').ab,kw,ti. | 2057687 |
| 27 |  | OR 24-26 | 2909937 |
| 28 | Study type | exp Consensus/ | 75713 |
| 29 |  | exp Consensus Development/ | 24880 |
| 30 |  | exp Practice Guideline/ | 579489 |
| 31 |  | ‘Health Planning Guideline’.ti,ab,kw. | 2 |
| 32 |  | (guideline or practice guideline or consensus development conference or consensus development conference, NIH).ti,ab,kw. | 100407 |
| 33 |  | (position statement* or policy statement* or practice parameter* or best practice*).ti,ab,kw. | 51923 |
| 34 |  | (standards or guideline or guidelines).ti,ab,kw. | 159319 |
| 35 |  | ((practice or treatment* or clinical) adj guideline*).ab. | 64569 |
| 36 |  | (CPG or CPGs).ti. | 7082 |
| 37 |  | consensus*.ti,kw. | 35153 |
| 38 |  | recommendat*.ti,kw. | 55504 |
| 39 |  | OR 28-38 | 858890 |
| 40 | N/A | 4 AND 27 AND 39 | **1207** |
